# Intraoperative pivot-shift accelerometry combined with anesthesia improves the measure of rotatory knee instability in anterior cruciate ligament injury

**DOI:** 10.1186/s40634-021-00396-1

**Published:** 2021-09-24

**Authors:** Gastón Caracciolo, Roberto Yáñez, Rony Silvestre, Carlos De la Fuente, Héctor Zamorano, Alejandra Ossio, Lars Strömbäck, Sebastian Abusleme, Felipe P. Carpes

**Affiliations:** 1 Clínica MEDS, Santiago, RM 7691236 Chile; 2 Centro de investigación en Medicina, Ejercicio, Deporte y Salud, Clínica MEDS, Santiago, RM 7691236 Chile; 3 Applied Neuromechanics Research Group, Laboratory of Neuromechanics, Federal University of Pampa, Uruguaiana, RS 97500-970 Brazil; 4 Carrera de Kinesiología, Departamento de Cs. de la Salud, Facultad de Medicina, Pontificia Universidad Católica de Chile, Santiago, RM 7820436 Chile

**Keywords:** Joint laxity, Joint stability, Ligaments, Kinematics, Lower extremity, Rupture

## Abstract

**Purpose:**

The knee stiffness acquired following an Anterior Cruciate Ligament (ACL) injury might affect clinical knee tests, i.e., the pivot-shift maneuver. In contrast, the motor effects of spinal anesthesia could favor the identification of rotatory knee deficiencies prior to ACL reconstruction. Hence, we hypothesized that the intra-operative pivot-shift maneuver under spinal anesthesia generates more acceleration in the lateral tibial plateau of patients with an injured ACL than without.

**Methods:**

Seventy patients with unilateral and acute ACL rupture (62 men and 8 women, IKDC of 55.1 ± 13.8 pts) were assessed using the pivot-shift maneuver before and after receiving spinal anesthesia. A triaxial accelerometer was attached to the skin between Gerdys’ tubercle and the anterior tuberosity to measure the subluxation and reduction phases. Mixed ANOVA and multiple comparisons were performed considering the anesthesia and leg as factors (alpha = 5%).

**Results:**

We found a higher acceleration in the injured leg measured under anesthesia compared to without anesthesia (5.12 ± 1.56 m.s^− 2^ vs. 2.73 ± 1.19 m.s^− 2^, *p* < 0.001), and compared to the non-injured leg (5.12 ± 1.56 m.s^− 2^ vs. 3.45 ± 1.35 m.s^− 2^, *p* < 0.001). There was a presence of significant interaction between leg and anesthesia conditions (*p* < 0.001).

**Conclusions:**

The pivot-shift maneuver performed under anesthesia identifies better rotatory instability than without anesthesia because testing the pivot-shift without anesthesia underestimates the rotatory subluxation of the knee by an increased knee stiffness. Thus, testing under anesthesia provides a unique opportunity to determine the rotational instability prior to ACL reconstruction.

## Introduction

The anterior cruciate ligament (ACL) rupture has an incidence of 68.6 per 100,000 people-years [31], and most cases (~ 80%) involve rotatory instability [3, 25]. Clinically, the rotatory instability is tested through the pivot-shift maneuver [30], and it has high specificity for ACL insufficiency [12] and correlates well with self-reports of knee function [20]. However, the altered activity of gamma motor neurons may increase knee stiffness after an ACL rupture [17, 25]. Hence, the knee rotation in patients with an ACL injury may be restricted.

When the ACL is insufficient during the pivot-shift maneuver, the lateral tibial plateau displaces more anteriorly and medially (subluxation) followed by the stretching of the iliotibial band [3]. Because of that, a sudden higher posterior and lateral shift of the lateral tibial plateau (reduction) is caused [11, 16]. Unfortunately, this joint movement relates to residual knee instability, mainly triggered during cutting, twisting, or pivoting motions [34]. In consequence, the scintigraphy activity in the subchondral bone and the risk of early knee osteoarthrosis increase [34].

On the other hand, meniscal injuries (bucket handle meniscus tear), collateral ligamentous laxity, or the quality of pivot-shift execution among clinicians can increase a false-negative diagnosis (type II error) of rotatory knee instability [17, 25, 26]. This false-negative diagnosis can affect clinical decisions. For example, the pertinence of an extraarticular anterolateral tenodesis could be affected if there is a false-negative rotatory instability [6, 22]. Thus, the improvement of pivot-shift execution has an important clinical significance.

Regarding the last improvements of the test, the pivot-shift maneuver has added accelerometry measures to quantify the rotatory knee laxity [1, 22]. However, applying the pivot-shift accelerometry for a better rotatory knee laxity detection after an ACL injury remains unclear. As anesthesia can selectively reduce the motor response of skeletal muscles, here we hypothesized that the intra-operative pivot-shift maneuver under spinal anesthesia generates more acceleration in the lateral tibial plateau of patients with an injured ACL than without. Therefore, the goal of our study was to determine the acceleration of the lateral tibial plateau during the pivot-shift maneuver in patients with an acute ACL injury under and without spinal anesthesia.

## Methods

### Patients

Seventy patients (62 men and 8 women) diagnosed with an acute ACL rupture were recruited between 2019 and 2020. Table 1 presents the demographic variables of the sample.

**Table 1:** Basal demographic characteristics of the patients

	*mean ± SD*	[min - max]
Age *(years)*	30.8 ± 10.4	[20–50]
Height *(m)*	1.73 ± 0.08	[1.62–1.77]
Body mass *(kg)*	78.2 ± 12.6	[65.1–86.5]
BMI (kg*m^*− 2*^*)*	26.1 ± 3.6	[23.0–28.7]
IKDC score *(pts)*	55.1 ± 13.8	[50.6–71.2]

*BMI* Body mass index, *SD* Standard deviation, *IKDC* International Knee Documentation Committee score

The inclusion criteria were: i) unilateral and acute ACL rupture (low edema and flexion range of motion of at least 90° within the first 3 weeks after injury) [9]; ii) positive Lachman and draw tests, iii) sports non-contact ACL-rupture [2]; iv) direct signs of ACL rupture in the magnetic resonance imaging [4]; v) arthroscopy confirmation of the ACL-rupture [25, 36]; vi) age between 20 and 50 years old; and vii) diagnosis and surgery conducted at the MEDS clinic (Santiago, Chile). The exclusion criteria were: i) ACL re-rupture; ii) presence of associated injuries (bone, ligament, or meniscus [10, 15]; iii) the presence of a previous orthopedic condition (patellofemoral dysfunction, anterior knee pain, leg length differences, knee varus or valgus, or osteoarthritis); iv) not being eligible for anesthesia [13]; v) rheumatological and metabolic conditions; vi) neurological conditions; vii) active infection; and viii) cognitive impairment. All cases satisfying the admissibility criteria provided written consent to participate. The study was approved by the institutional ethics board from MEDS clinic (Santiago, Chile) and conducted according to the Declaration of Helsinki.

### ACL rupture diagnosis

A senior orthopedic medical doctor Roberto Yañez confirmed the ACL rupture through a combination of clinical history, physical exam, magnetic resonance images, and arthroscopy. An acute unilateral rupture of the ACL was fully diagnosed when patients indicate a sport non-contact ACL rupture mechanism (whether in flexion, abduction, internal rotation or any combinations) [18], positive signs for acute inflammation, a positive Lachman and anterior draw test [12], and the non-integrity of ACL determined by magnetic resonance imaging [4].

### Experiment

The pivot-shift testing [24] was performed before and after the anesthesia delivery, with the patient lying supine position on an operating table. A disinfected wireless accelerometry was attached over the lateral tibial plateau to acquire the accelerometry data when the pivot-shift maneuver was executed. The same senior orthopedic surgeon performed the pivot-shift maneuver in all patients. The orthopedic surgeon had more than 30 years of experience with regular use of the test and a minimum of 100 ACL reconstructions per year in a regular year.

Prior to ACL reconstruction, spinal anesthesia was applied to patients receiving bupivacaine (10 to 12 mg) and intravenous sedation together with fentanyl 20 μg, while receiving oxygen via a facemask to ensure 98% oxygen saturation. Non-invasive monitors were used for electrocardiography, blood pressure, and pulse oximeter monitoring [13]. The femoral nerve block for bone-to-bone patellar allograft and a saphenous nerve block for semitendinosus-gracilis allograft was performed guided by ultrasonography [13]. The effect of the anesthesia on the quadriceps-hamstring strength was checked by the surgeon performing manual strength testing. A second pivot-shift maneuver was executed when the quadriceps strength reached an M-score equal to zero (non-visible or palpable muscle contraction). Finally, the accelerometer was detached, and the cleaning and sterilization procedures were performed to proceed with the ACL reconstruction.

### Pivot-shift testing and accelerometry measurement

**Fig. 1:**
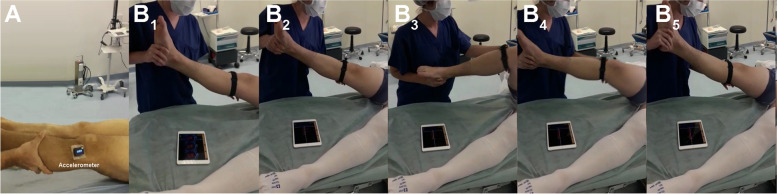
Intra-operative pivot-shift maneuver before surgery. **A** Sensor orientation. **B1** to **B5** The sequence of the pivot-shift test

The pivot-shift test was repeated five times under conditions of anesthesia and non-anesthesia on both injured and non-injured legs. The movement was tracked by a triaxial accelerometer (Trigno™ accelerometry system, Delsys Inc., USA) with 3 degrees of freedom, range of ±3 g, sampling resolution of 20 ± 5 Hz, > 40 dB*dec − 1 and 450 ± 50 Hz > 80 dB*dec^− 1^, basal noise (RMS) of 0.016 g for a range of ±1.5 g and 0.032 g for a range of ±6 g, a bandwidth of DC − 50 ± 5 Hz with 20 dB*dec^− 1^, offset error of ±0.21 g for the X-axis and − 0.42 g for the Z-axis, and an accelerometer resolution depth of 10 bits [8]. The accelerometer was placed in the middle point between the patient’s Gerdy’s tubercle and the tibial tuberosity, with the patient in a supine position [1]. The accelerometer was aligned to the longitudinal axis of the tibia bone [1]; see Fig. 1.

**Fig. 2:**
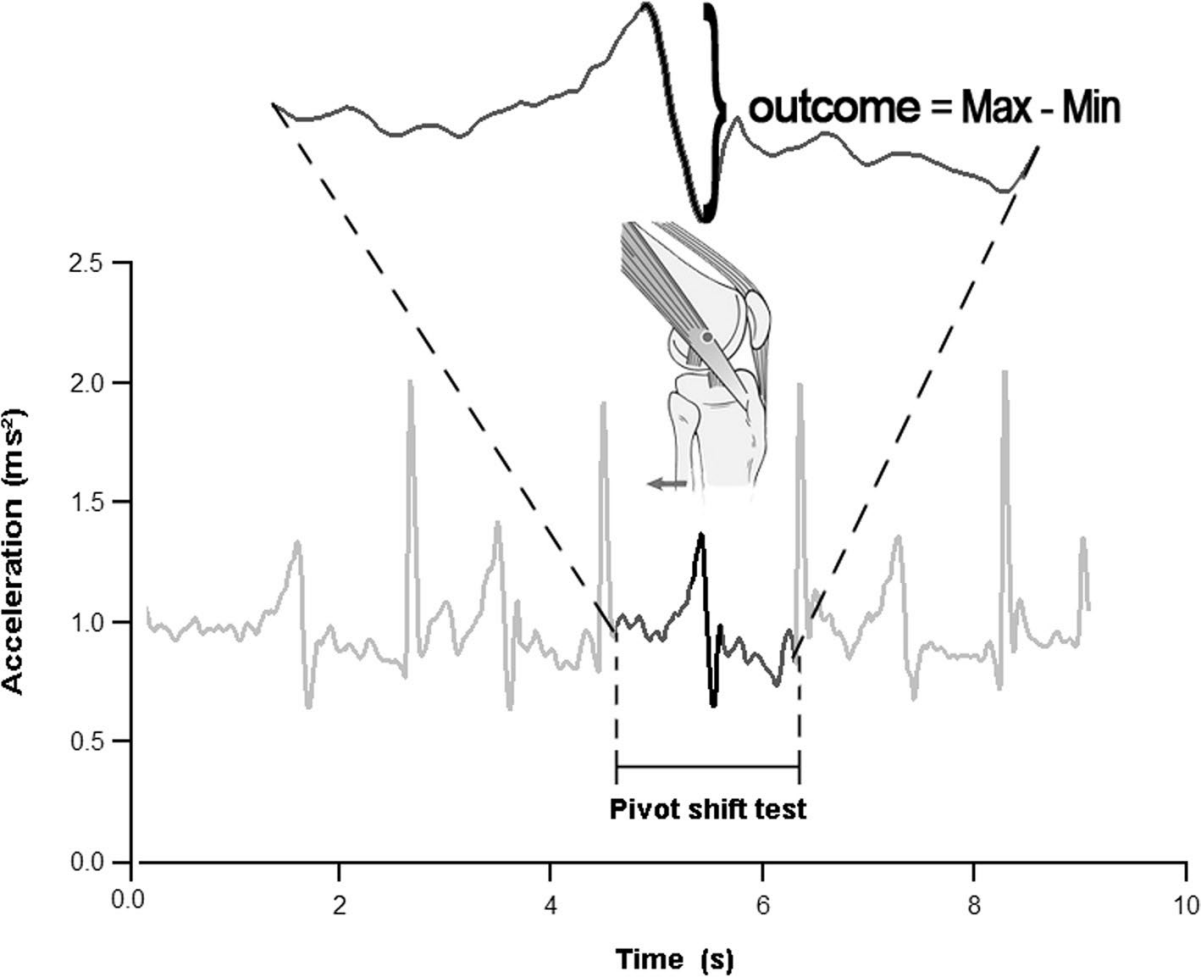
The acceleration measured during the pivot-shift maneuver resulting in a sudden posterior displacement of the lateral tibial plateau in the reduction phase

The variable of interest was the difference between the maximal and minimum value of the resultant acceleration on the lateral aspect of the tibia during each pivot-shift maneuver (Fig. 2). We chose the delta acceleration of the resultant acceleration to obtain a physical measure that can transduce equally the flexion, valgus, and medial rotation induced by the clinical test. Furthermore, the mean of five repetitions was used to attenuate the possible outlier accelerations obtaining an averaged pattern. Hence, the resultant acceleration was obtained from $\boldsymbol{a}=\sqrt[]{{{\boldsymbol{a}}_{\boldsymbol{x}}}^{\mathbf{2}}+{{\boldsymbol{a}}_{\boldsymbol{y}}}^{\mathbf{2}}+{{\boldsymbol{a}}_{\boldsymbol{z}}}^{\mathbf{2}}}$ measured in m*s^− 2^. Thus, the acceleration pattern of each pivot-shift maneuver was clearly observed during the data acquisition. For methodological details, see Figs. 1 and 2.

### Statistical analysis

Data were reported as the mean and standard deviation. The Kolmogorov-Smirnov test confirmed the normality of data distribution. The data homoscedasticity was confirmed using Levene’s test, and sphericity was confirmed using Mauchly’s test. The main effects for anesthesia and injury, and possible interactions, were determined using a mixed-ANOVA of two factors 2 × 2 (anesthesia factor with two levels: without and under anesthesia; and injury factor with two levels: injured and non-injured leg). A two-tailed paired t-test was applied to identify paired differences for repeated measures and a two-tailed independent t-test for independent data. The partial-eta squared was interpreted as small (> 0.01–0.06), moderate (> 0.06–0.14), and large (> 0.14) [27]. The Cohen’s d effect was interpreted as small (> 0.2–0.5), moderate (> 0.5–0.8), and large (> 0.8) [27]. The statistical significance was set at 0.05 for all analyses performed using the statistical software SPSS 26 (IBM Corp., USA). The post-hoc power analysis was described and estimated using the G*Power 3.1.9.2 software (Kiel & Dusseldorf University, Germany).

## Results

**Fig. 3:**
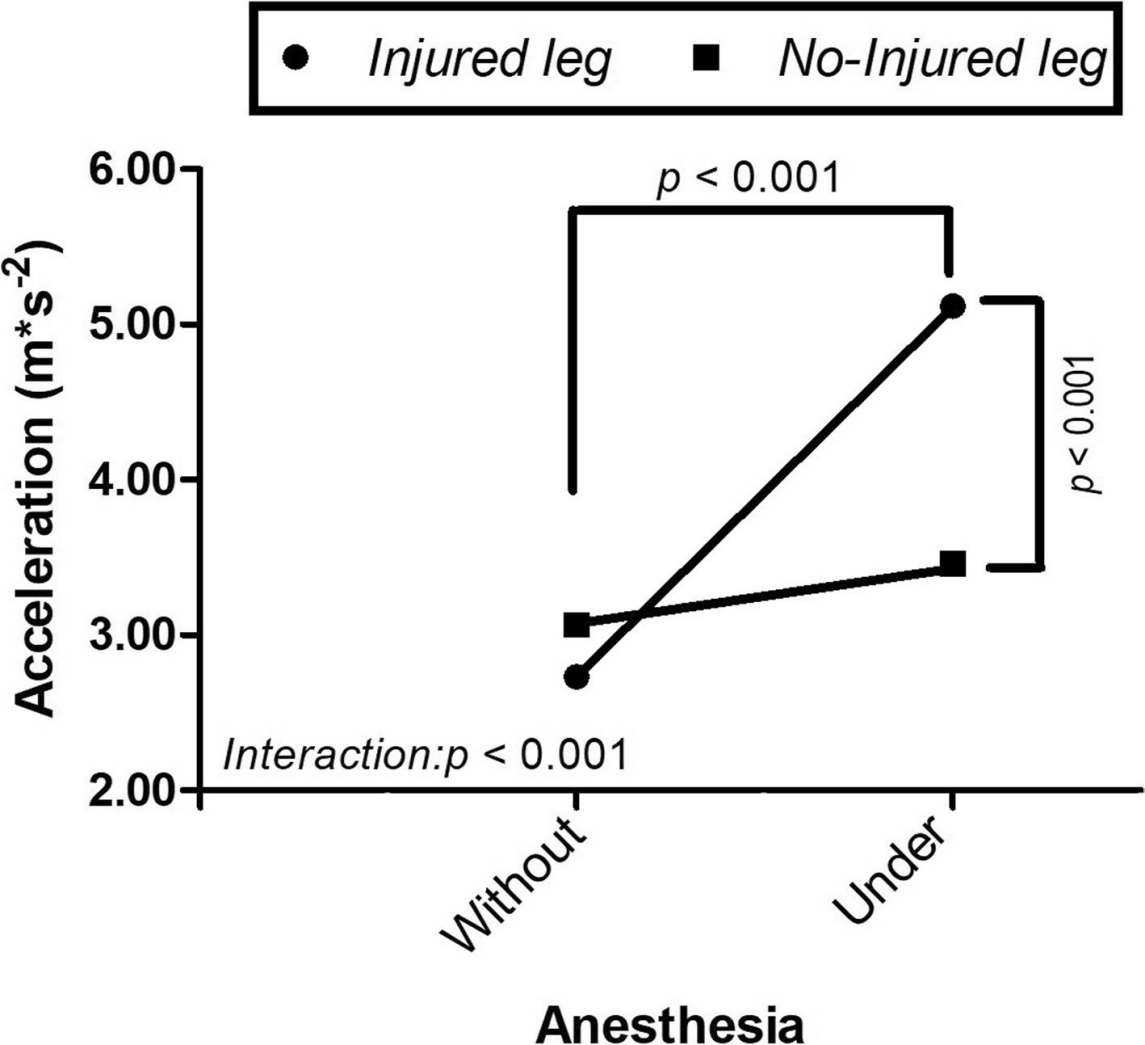
Intra-operative pivot-shift assessment using an accelerometer in the lateral tibial plateau without and under anesthesia in patients with injured Anterior Cruciate Ligament

A main effect was found for anesthesia (F_(1,69)_ = 16.3, *p* < 0.001, and ŋ^2^ = 0.106; moderate effect size) and for injury factors (F_(1,69)_ = 82.4, *p* < 0.001, and ŋ^2^ = 0.374; large effect size). There was an interaction between anesthesia and injury factors (F_(1,69)_ = 36.8, *p* < 0.001, and ŋ^2^ = 0.211; large effect size). See Fig. 3 for details.

Acceleration was higher when the pivot-shift maneuver was performed under anesthesia compared to without anesthesia in the injured leg (5.12 ± 1.56 m.s^− 2^ vs. 2.73 ± 1.19 m.s^− 2^, Δ = 2.39 m·s^− 2^, *p* < 0.001, t_(1,69)_ = − 10.32, d = 1.72; large effect size). In the injured leg the acceleration was also higher compared to the non-injured leg under anesthesia (5.12 ± 1.56 m.s^− 2^ vs. 3.45 ± 1.35 m.s^− 2^, Δ = 1.67 m.s^− 2^, *p* < 0.001, t_(1,69)_ = 6.76; d = 1.66, large effect size), see Fig. 3 and Table 2 for details. The post-hoc power analysis obtained was 0.98 for an *n* = 70.

**Table 2:** Accelerometry of lateral tibial plateau during the pivot-shift maneuver in patients with ACL injury

	**Without anesthesia** *mean ± SD* (m*s^*− 2*^*)* *n* = 70	**Under anesthesia** *mean ± SD* (m*s^*− 2*^*)* *n* = 70	**Within-group Δ** *Under – Without* (m*s^*− 2*^*)* *n* = 70
***ACL ruptured leg***			
Injured leg	2.73 ± 1.19^*^	5.12 ± 1.56^*, λ^	2.39
No injured leg	3.07 ± 1.20	3.46 ± 1.33^***λ***^	0.39

*ACL* Anterior Cruciate Ligament, *SD* Standard Deviation, *Δ* Differences

*Within-group statistical significance *p* < 0.001

**λ**Between-group statistical significance *p* < 0.001

## Discussion

In this study, we demonstrated that the pivot-shift maneuver conducted without spinal anesthesia generates lower acceleration of the lateral tibial plateau during the reduction phase than under anesthesia in ACL injury. This suggests that false-negative outcomes may be induced during the pivot-shift maneuver for ACL injury conducted without anesthesia. Accordingly, we recommend assessing the pivot-shift maneuver under anesthesia. We highlight that the use of accelerometry performed by the same clinician adds sensitivity to identify rotatory instability and diminishes subjective appreciations. Knowing residual rotatory instability tested under anesthesia without the increased co-activation of the hamstrings and quadriceps is crucial to choosing a better surgical and clinical management.

Several factors can increase the activity of the hamstrings in patients with ACL injury resulting in an attenuated pivot-shift sign, as we observed in this study. It is known that the pivot-shift maneuver can induce knee joint instability, but this is typically controlled in healthy knees without disproportionated increasing knee muscle stiffness [3]. However, the maneuver may induce hamstrings activation to restrict the knee rotation and displacement [32]. The anterior displacement and rotatory instability movement caused by the pivot-shift maneuver excites muscle spindles by stretching stimulus, eliciting hamstring activation in an involuntary attempt to restore joint stability [32].

Also, there is a neural link between ligaments and spindles, where mechanical changes in stress over the ligaments triggers muscle spindle activity [32]. Hence, it is highly probable that both the ruptured and non-ruptured ACL may induce the activity of hamstrings during testing to counteract the anterior tibial displacement and medial knee rotation [5]. As a consequence, our results agree with the muscle-ligament-reflex phenomena described by Solomonow and Krogsgaard [19, 23, 32]. This increased neuromuscular response of knee muscles is supported by the reduced acceleration found in the injured ACL patients without anesthesia and by the increased tibial acceleration during the subluxation phase of the pivot-shift maneuver when the anesthesia is delivered, blocking the nerve innervation of hamstrings. Thus, the motor effects of the anesthesia are clinically useful to determine the residual rotatory instability in ruptured ACL.

Unfortunately, a hiding pivot-shift sign results in early osteoarthrosis [34], and an instability sensation can often remain in patients [21]. The fact that rotational instability (positive pivot shift sign) may be an important precursor of knee osteoarthrosis [7], supports the efforts that minimize the false-negative rotatory knee laxity. A recent cadaveric study with sectioned ACL has found that rotational instability is not adequately resisted by intact anterolateral ligaments or the iliotibial band [28]. When both structures are sectioned, 7 out of 10 knees developed high rotational instability [28]. In the long-term, this unrestricted pivoting movement is associated with knee osteoarthrosis [14]. Unfortunately, patients can hide symptoms [35], showing persistent joint movement alterations after the ACL reconstruction [33]. These changes and consequences reaffirm the importance of implementing better alternatives to measure rotatory knee instability.

Regarding the false-positive diagnosis of rotatory instability, muscle activation and stiffness are important factors in assessing injured ACL patients. Indeed, exploring the positive pivot-shift sign should encourage clinicians and researchers. Thus, performing the pivot-shift maneuver with patients under anesthesia is a unique opportunity to determine the rotational knee deficits in ACL ruptured patients. We could mention the reproducibility of the pivot-shift maneuver as a relevant limitation, such as it has been described previously by the literature [26]. Also, we believe that accelerometers are a good measurement tool but not the best for assessing dynamic knee laxity [29].

## Conclusions

The pivot-shift maneuver executed without anesthesia elicits lower acceleration of the tibial plateau during the reduction phase than under anesthesia due to increased knee stiffness. Hence, performing the pivot-shift maneuver executed under anesthesia permitted a more sensitive identification of rotational knee laxity.

## Data Availability

Data will be available after publication in an external repository. The external link is: https://www.researchgate.net/publication/354628903_Supplementary_data_Intraoperative_pivotshift_accelerometry_combined_with_anesthesia_improves_the_measure_of_rotatory_knee_instability_in_anterior_cruciate_ligament_injury.
